# PET/MR in recurrent glioblastoma patients treated with regorafenib: [^18^F]FET and DWI-ADC for response assessment and survival prediction

**DOI:** 10.1259/bjr.20211018

**Published:** 2021-11-23

**Authors:** Giuseppe Lombardi, Alessandro Spimpolo, Sara Berti, Cristina Campi, Maria Giulia Anglani, Rossella Simeone, Laura Evangelista, Francesco Causin, Giovanni Zorzi, Giancarlo Gorgoni, Mario Caccese, Marta Padovan, Vittorina Zagonel, Diego Cecchin

**Affiliations:** 1 Department of Oncology, Oncology 1, Veneto Institute of Oncology – IRCCS, Padua, Italy; 2 Nuclear Medicine Unit, Department of Medicine - DIMED, Padua University Hospital, Padua, Italy; 3 Department of Mathematics, University of Genoa, Genoa, Italy; 4 Neuroradiology Unit, Azienda Ospedaliera di Padova, Padua, Italy; 5 Department of Neurosciences (DNS), University of Padua, Padua, Italy; 6 Radiopharmacy, Sacro Cuore Don Calabria Hospital, Negrar, Verona, Italy

## Abstract

**Objective:**

The use of regorafenib in recurrent glioblastoma patients has been recently approved by the Italian Medicines Agency (AIFA) and added to the National Comprehensive Cancer Network (NCCN) 2020 guidelines as a preferred regimen. Given its complex effects at the molecular level, the most appropriate imaging tools to assess early response to treatment is still a matter of debate. Diffusion-weighted imaging and O-(2-^18^F-fluoroethyl)-L-tyrosine positron emission tomography ([^18^F]FET PET) are promising methodologies providing additional information to the currently used RANO criteria. The aim of this study was to evaluate the variations in diffusion-weighted imaging/apparent diffusion coefficient (ADC) and [^18^F]FET PET-derived parameters in patients who underwent PET/MR at both baseline and after starting regorafenib.

**Methods:**

We retrospectively reviewed 16 consecutive GBM patients who underwent [^18^F]FET PET/MR before and after two cycles of regorafenib. Patients were sorted into stable (SD) or progressive disease (PD) categories in accordance with RANO criteria. We were also able to analyze four SD patients who underwent a third PET/MR after another four cycles of regorafenib. [^18^F]FET uptake greater than 1.6 times the mean background activity was used to define an area to be superimposed on an ADC map at baseline and after treatment. Several metrics were then derived and compared. Log-rank test was applied for overall survival analysis.

**Results:**

Percentage difference in FET volumes correlates with the corresponding percentage difference in ADC (*R* = 0.54). Patients with a twofold increase in FET after regorafenib showed a significantly higher increase in ADC pathological volume than the remaining subjects (*p* = 0.0023). Kaplan–Meier analysis, performed to compare the performance in overall survival prediction, revealed that the percentage variations of FET- and ADC-derived metrics performed at least as well as RANO criteria (*p* = 0.02, *p* = 0.024 and *p* = 0.04 respectively) and in some cases even better. TBR Max and TBR mean are not able to accurately predict overall survival.

**Conclusion:**

In recurrent glioblastoma patients treated with regorafenib, [^18^F]FET and ADC metrics, are able to predict overall survival and being obtained from completely different measures as compared to RANO, could serve as semi-quantitative independent biomarkers of response to treatment.

**Advances in knowledge:**

Simultaneous evaluation of [^18^F]FET and ADC metrics using PET/MR allows an early and reliable identification of response to treatment and predict overall survival.

## Introduction

Glioblastoma multiforme (GBM), the most common primary malignant brain tumor in adults, still carries a dismal prognosis, with a median overall survival of less than 24 months, even after maximal safe resection, concomitant chemoradiotherapy and adjuvant temozolomide.^1–3^ In the setting of disease relapse, the use of regorafenib has been recently approved by the Italian Medicines Agency (AIFA) and added to the new National Comprehensive Cancer Network (NCCN) 2020 guidelines as a preferred regimen, based on promising results from a multicenter Phase II trial (REGOMA) comparing this new drug with the standard lomustine regimen.^4^ Regorafenib is an orally available multi kinase inhibitor with several molecular targets involved in angiogenesis (VEGFR1-3 and TIE2), oncogenesis (KIT, RET, RAF1, and BRAF) and maintenance of the tumoral microenvironment (PDGFR and FGFR).^5–7^ Given the complexity of its effects at the molecular level, the choice of the most appropriate imaging parameters to be used with patients treated with regorafenib is still a matter of debate. Currently, the recommendations of the Response Assessment in Neuro-Oncology (RANO) Study Group are widely used in both clinical practice and research settings and were also implemented in the REGOMA trial.^4,8^ The RANO criteria are based on measurement of areas of contrast-enhancement on post-gadolinium *T*_1_ weighted sequences and of non-enhancing disease captured on *T*_2_ weighted/fluid attenuated inversion recovery (FLAIR) images. This approach, however, has already been shown to have several limitations and shortcomings in patients treated with anti angiogenetic drugs, such as bevacizumab, given the normalization of vascular permeability and the related decrease in contrast enhancement induced by these agents.^8–10^ In fact, up to 40% of patients treated with bevacizumab show seemingly stable contrast-enhancing disease with an increase in *T*_2_ weighted/FLAIR signal abnormalities, indicating disease progression.^11^ Moreover, the lack of a quantifiable measure of non-enhancing disease progression, and the confounding effect of radiation therapy, ischemic injury, and post-operative changes on FLAIR images further complicate the issue.

Diffusion-weighted imaging (DWI) is a promising methodology that could improve the assessment of treatment response in GBM, thereby extending the existing RANO criteria.^12^ It is based on measuring the Brownian motion of water molecules and the various constraints that hamper this physical phenomenon in live tissues. Moreover, DWI-derived apparent diffusion coefficient (ADC) maps offer quantitative information related to tumor cellularity and have already been used in glioma patients to detect the presence of neoplastic tissue in the peritumoral edema.^13,14^ Since necrosis, ischemia and inflammation are known to influence water diffusion, heterogeneous ADC values are usually evident in tumoral areas, especially after treatment.^15,16^ Consequently, the mean ADC values of one area can fail to depict the spatial heterogeneity of brain tumors, although histogram analysis has already been successfully used as a possible workaround.^16,17^

O-(2-^18^F-fluoroethyl)-L-tyrosine (^18^F–FET), an amino acid tracer used in positron emission tomography (PET), is another important tool routinely used for therapy assessment during anti-angiogenetic treatment.^18,19^ Even though several studies have already demonstrated the additional value of amino acid PET over conventional MR-based assessment in this setting,^20,21^ the interplay between ADC and [^18^F]FET PET in patients treated with regorafenib has been explored so far only in a small case series comprising five cases.^22^

The aim of this study was to evaluate the variations in DWI/ADC- and [^18^F]FET PET-derived parameters in recurrent patients undergoing PET/MR both at baseline and after beginning regorafenib. Furthermore, we analyzed the performance in survival prediction of RANO criteria compared to DWI/ADC- and [^18^F]FET PET-derived parameters.

## Methods and materials

This was a single-center, retrospective, observational study conducted in accordance with the Declaration of Helsinki and after formal approval by our local Ethics Committee (protocol number: AOP1673 - 4831/AO/20). All patients gave written informed consent before undergoing the [^18^F]FET PET/MR, including access to their data for research purposes.

### Patient selection

Among 52 patients treated with regorafenib, we retrospectively selected 16 consecutive recurrent GBM patients who underwent [^18^F]FET PET/MR from May 2019 to October 2020 at the Nuclear Medicine Unit of Padua University Hospital before and after two cycles of regorafenib; 4/16 patients were followed up with a third PET/MR; all of the patients were treated at the Veneto Institute of Oncology-IRCCS in Padua. Excluded Patients were those who underwent [^18^F]FET PET/MR but lacked one of the following inclusion criteria:

1. Histologically confirmed glioblastoma.
2. Radiologically and/or histologically confirmed disease relapse after conventional treatment according to RANO criteria (maximal safe resection followed by chemoradiotherapy).
3. Acquisition of baseline [^18^F]FET PET/MR no sooner than 1 week before starting regorafenib.
4. Acquisition of a second [^18^F]FET PET/MR no later than 2 weeks after two cycles of regorafenib.
5. No treatment changes between baseline and post-regorafenib [^18^F]FET PET/MR.

### Image acquisition and reconstruction

All [^18^F]FET PET/MR images were acquired with a 3 T Biograph integrated PET/MR scanner (Siemens Healthcare, Germany) at the Nuclear Medicine Unit of Padua University Hospital, Italy. Following the most recent recommendations by the European Association of Nuclear Medicine, all study patients were required to fast for a minimum of 4 h before the intravenous administration of approximately 250 MBq of ^18^F–FET. Dynamic PET data were acquired from the time of tracer administration to 50 min post-injection,^23^ while at the same time a standardized MR protocol was performed. The latter included: 1 mm isotropic 3D *T*_1_ weighted magnetization-prepared rapid acquisition gradient echo (MPRAGE) (TR 2400 ms, TE 3.24 ms, slice thickness 1 mm, matrix size 256 × 256, FOV 256 × 256 mm) before and after contrast enhancement, 3D isovolumetric FLAIR (TR 5000 ms, TE 394 ms, TI 1800 ms, slice thickness 1 mm, matrix size 256 × 256, FOV 250 × 250 mm) and RESOLVE^®^ sequence (Siemens Healthcare, Germany) (TR 5,000 ms, TE1 72 ms, TE2 122 ms, voxel size 1.56 × 1.56 x 3.12 mm), a high-resolution DWI sequence based on a readout-segmented echoplanar imaging (EPI) strategy.^24^ ADC images were calculated from acquired DWI images with a b-value of 1000 s/mm^2^ and 0 s/mm^2^. The contrast medium used with all patients was gadobutrol 0.1 mmol/Kg (Gadovist^®^, Bayer Inc., Mississauga, Ontario).

A reconstruction of single frame PET images obtained at 20–40 min after tracer injection was used for the present study as suggested by EANM guidelines. Although kinetic analysis (in particular, the analysis of the time to peak of tracer uptake) could be predictive of response to treatment,^25^ the analysis of the 50 min dynamic curve pattern is beyond the scope of the present paper that aims at comparing static PET indexes with RANO criteria. Standard corrections for decay, scatter and dead time were performed. A clinical UTE sequence (Siemens Healthcare, Germany) was included in the MR protocol (because more advanced AC methods are limited to a research setting and not directly applicable to a standard clinical setting) and used for attenuation correction of PET. The quality of the derived UTE map was visually assessed in all patients. The PET data were reconstructed using a 3D ordered subset expectation maximization algorithm with 8 iterations, 21 subsets and a 3 mm Gaussian filter, from which PET images with a 256 × 256 matrix size (voxel size = 2.32 × 2.32 × 2.03 mm) were derived.

### Qualitative image analysis

One neuroradiologist and one nuclear medicine physician (with 7 and more than 10 years’ experience in the field of neuro-oncology, respectively), blind to the patients clinical outcomes and the follow-up imaging, jointly reviewed all [^18^F]FET PET/MR images at both baseline and post-regorafenib. The MR portion of the study have been evaluated first (comparing baseline MR and post-regorafenib MR) blinded to PET results. In accordance with the latest Response Assessment Criteria for High-Grade Gliomas by the Response Assessment in Neuro-Oncology (RANO) Working Group,^8,26^ study patients were divided into the following response-assessment categories: Complete Response (CR), Partial Response (PR), Stable Disease (SD), Progressive Disease (PD).

In cases of assumed CR or PR at the post-regorafenib time point, a follow-up MR scan was performed at least 4 weeks later and reviewed for confirmation.

PD was defined (according to RANO) as the fulfillment of one or more of the following conditions:

1. ≥ 25% increase in the sum of the products of the perpendicular diameters of the enhancing lesions compared with the smallest tumor measurement at baseline;
2. appearance of any new contrast-enhancing lesion;
3. significant increase in T2/FLAIR non-enhancing lesion.

Patients fell into the SD category if they did not meet the conditions for CR, PR, or PD, and were administered the same or a lower dose of corticosteroids.

### Image data processing

The images were imported into PMOD (PMOD^®^ Technologies LLC, Zurich, Switzerland) for volume of interest (VOI) delineation.

[^18^F]FET PET (FET), post-contrast 3D *T*_1_ weighted MPRAGE (MDC) and ADC images were rigidly aligned to the pre-contrast 3D *T*_1_ weighted MPRAGE (T1).

The mean standardized uptake value of a crescent-shaped VOI (BG_FET_), manually drawn in the hemisphere contralateral to the tumor, was used as the [^18^F]FET background.^27^ The pathological FET volume (FET_vol/pat_) was segmented through a 3D semiautomatic contouring process, excluding areas with an [^18^F]FET uptake less than 1.6 times the background mean activity. This threshold was based on an [^18^F]FET biopsy-controlled study, where it was proven to accurately differentiate between tumoral and non-tumoral tissue.^28^ The chosen cut-off has been subsequently used successfully in a number of publications presenting histopathological confirmation and/or MR comparisons.^29,30^ The derived segmented volume was visually refined to exclude areas of non-specific [^18^F]FET spillover (major blood vessels, cranial bones, meninges etc.) using the aligned MDC images as the morphological reference.

**Figure 1.**
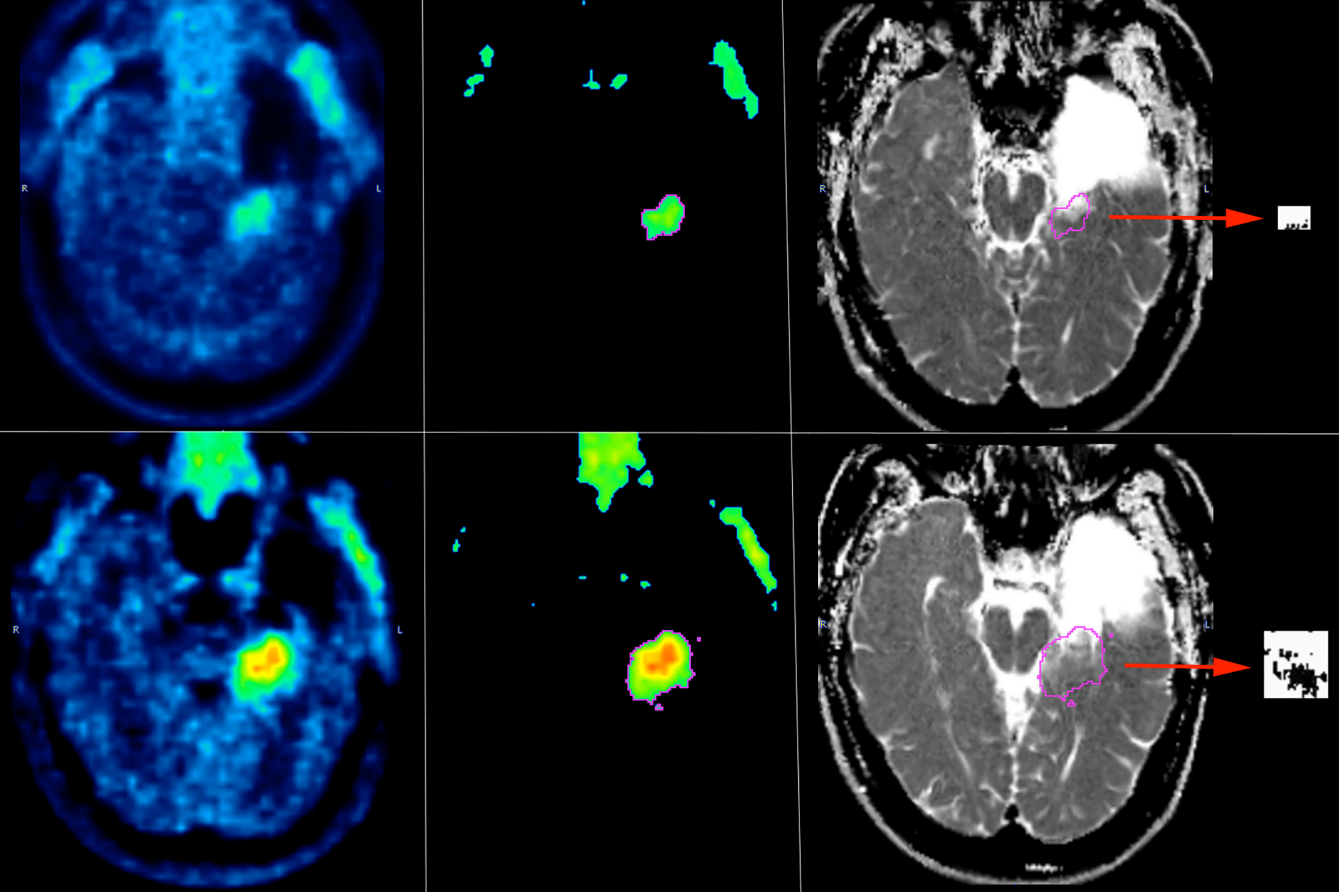
Overview of the methodology used to segment the pathological [^18^F]FET and ADC volumes in one of the study patients before (upper row) and after (lower row) treatment with regorafenib. Left: native [^18^F]FET PET images showing an area of pathological radiotracer uptake in the left mesial temporal lobe; center: the segmented pathological FET volume (FET_vol/pat_) determined through a 3D semiautomatic contouring process and excluding areas with [^18^F]FET uptake less than 1.6 times the mean background activity; right: the pathological FET volume superimposed onto ADC images and (in boxes pointed by red arrows) the resulting pixels (black dots) with values below the mean ADC background. [^18^F]FET, O-(2-^18^F-fluoroethyl)-L-tyrosine; ADC, apparent diffusion coefficient.

FET_vol/pat_ was then superimposed onto the ADC images (Figure 1) to obtain the corresponding ADC volume (ADC_vol_). The details are as follows:

1. FET_vol/pat_ was imported into the aligned ADC image.
2. Areas with non-specific high ADC values were subtracted (with the aim also to correct for anatomical distortions induced by metal implants and air filled cavities) from the original volume, pinpointing the ADC values in the cerebrospinal fluid of the lateral ventricles.
3. Areas of the original volume located outside the brain parenchyma were analogously subtracted.

A standard spherical volume (radius = 5 mm) was then placed on the ADC images in the hemisphere contralateral to the tumor, carefully avoiding lateral ventricles and major vessels, in order to derive the mean ADC value of the normal brain parenchyma (BG_ADC_). This method was chosen in view of the stability of the ADC values in the “healthy” brain parenchyma during treatment with antiangiogenetic agents.^12^ A qualitative assessment of the high resolution DWI and ADC derived maps was performed in every patient and revealed no significant distortions or misregistrations affecting the selected tumor area or background area.

The quality of alignment and segmentation was finally checked by an experienced nuclear medicine physician (with more than 10 years’ experience in the field of neuro-oncology).

### Data analysis

A pixel dump of FET_vol/pat_, BG_FET_, ADC_vol_, and BG_ADC_ was imported into the R software^31^ for further analyses. The mean ADC value of the BG_ADC_ was used as a threshold for ADC_vol_. Only those pixels below the threshold were considered pathologic (ADC_vol/pat_). The percentage differences in ADC_vol/pat_ and FET_vol/pat_ before and after regorafenib were calculated and compared (ΔADC_vol/pat_ = ADC_vol/pat_ (T1-T0)/T0 and ΔFET_vol/pat_ = FET_vol/pat_ (T1-T0)/T0).

### Statistical analysis

All statistical analyses were performed using the R Software. The Shapiro–Wilk normality test was performed on the distribution of all the parameters. Where normal distributions could not be assumed, non-parametric tests were performed. The percentage changes in ADC_vol/pat_ and FET_vol/pat_ before and after regorafenib were plotted and the Pearson correlation coefficient (PCC) calculated, assuming a linear correlation between the two variables. The differences in the percentage changes in FET_vol/pat_ and ADC_vol/pat_ between the response groups determined according to RANO criteria^8^ were compared using the Wilcoxon signed-rank test for repeated measures. The significance level (α) was set at 0.05. Log-rank test was applied for overall survival analysis. A *p*-value < 0.05 was considered significant.

## Results

### Patients

Our study population consisted of 15 IDH-wt and one glioblastoma NOS patients (6 females, 10 males, median age: 54.4 years, age range: 31–73 years). All the study patients had undergone maximal safe resection, adjuvant chemoradiotherapy with temozolomide and subsequent maintenance temozolomide (from 1 to 12 cycles) before relapsing. The median time elapsed between radiotherapy and baseline [^18^F]FET PET/MR was 319 days. In one subject, re-irradiation was given in a single fraction about 4 weeks before starting regorafenib. Eight of the sixteen patients had been surgically retreated before being scheduled for regorafenib, and at least 19 days passed before the first [^18^F]FET PET/MR was performed. All the study patients received two cycles of regorafenib (160 mg per day; 3 weeks on, 1 week off) without treatment interruption. The characteristics of the population are summarized in Table 1.

**Table 1.** Characteristics of the study population

Patient	Sex	Age	Histology	First treatment	Maintenance TMZ (cycles)	Second treatment before regorafenib	Latest surgery to first PET/MR (days)	Latest RT to first PET/MR (days)	First to second PET/MR (days)
**1**	M	40	GBM IDHwt	Surgery +RTCHT	6	Surgery	42	329	56
**2**	F	45	GBM IDHwt	Surgery +RTCHT	12		469	405	77
**3**	F	58	GBM IDHwt	Surgery +RTCHT	1	Surgery	62	1162	49
**4**	M	66	GBM IDHwt	Surgery +RTCHT	12		504	414	70
**5**	M	53	GBM IDHwt	Surgery +RTCHT	6		284	180	63
**6**	M	65	GBM IDHwt	Surgery +RTCHT	10		406	322	62
**7**	F	31	GBM IDHwt	Surgery +RTCHT	3		201	123	84
**8**	M	48	GBM IDHwt	Surgery +RTCHT	6	Surgery	41	858	70
**9**	F	39	GBM IDHwt	Surgery +RTCHT	6		363	312	56
**10**	F	48	GBM IDHwt	Surgery +RTCHT	8	Surgery	42	314	56
**11**	M	72	GBM IDHwt	Surgery +RTCHT	6	Surgery	55	333	56
**12**	M	60	GBM IDHwt	Surgery +RTCHT	2	Surgery	46	168	64
**13**	F	64	GBM IDHwt	Surgery +RTCHT	2		406	319	49
**14**	M	61	GBM IDHwt	Surgery +RTCHT	2	Surgery	19	183	56
**15**	M	48	GBM NOS	Surgery +RTCHT	12	Re-irradiation	3052	32	84
**16**	M	73	GBM IDHwt	Surgery +RTCHT	5	Surgery	36	239	48

GBM, glioblastoma; RTCHT, concomitant radio-chemotherapy; TMZ, temozolamide; wt, wildtype.

The 15th subject underwent re-irradiation (20 Gy, 1fr, EBRT) before starting regorafenib.

### [^18^F]FET PET/MR image analysis

**Figure 2.**
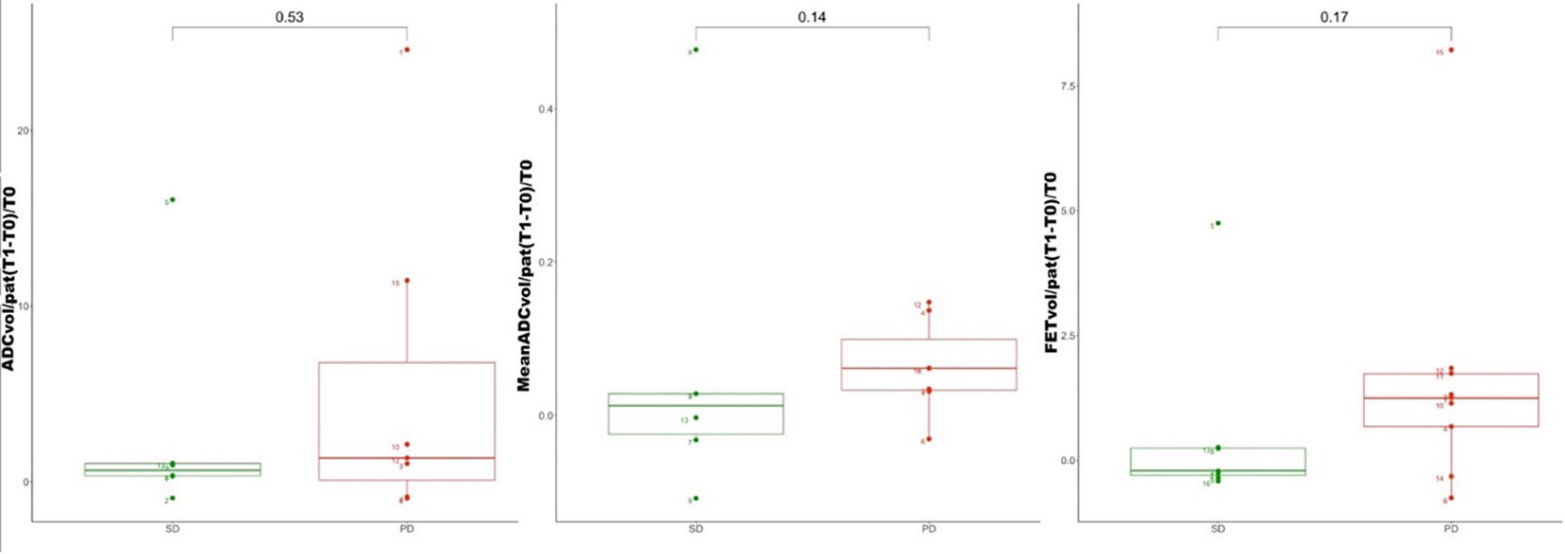
Variations in the ADC-derived parameters after regorafenib in study patients sorted into response groups according to RANO criteria. Left, the variation in pathological ADC volume (ADC_vol/pat_); center, the variation in the mean ADC values in the pathological ADC volume (mean ADC_vol/pat_); right, the variation in pathological FET volume (FET_vol/pat_); SD = Stable Disease; PD = Progressive Disease; T0 = First [^18^F]FET PET/MR; T1 = Second [^18^F]FET PET/MR; ADC, apparent diffusion coefficient.

**Figure 3.**
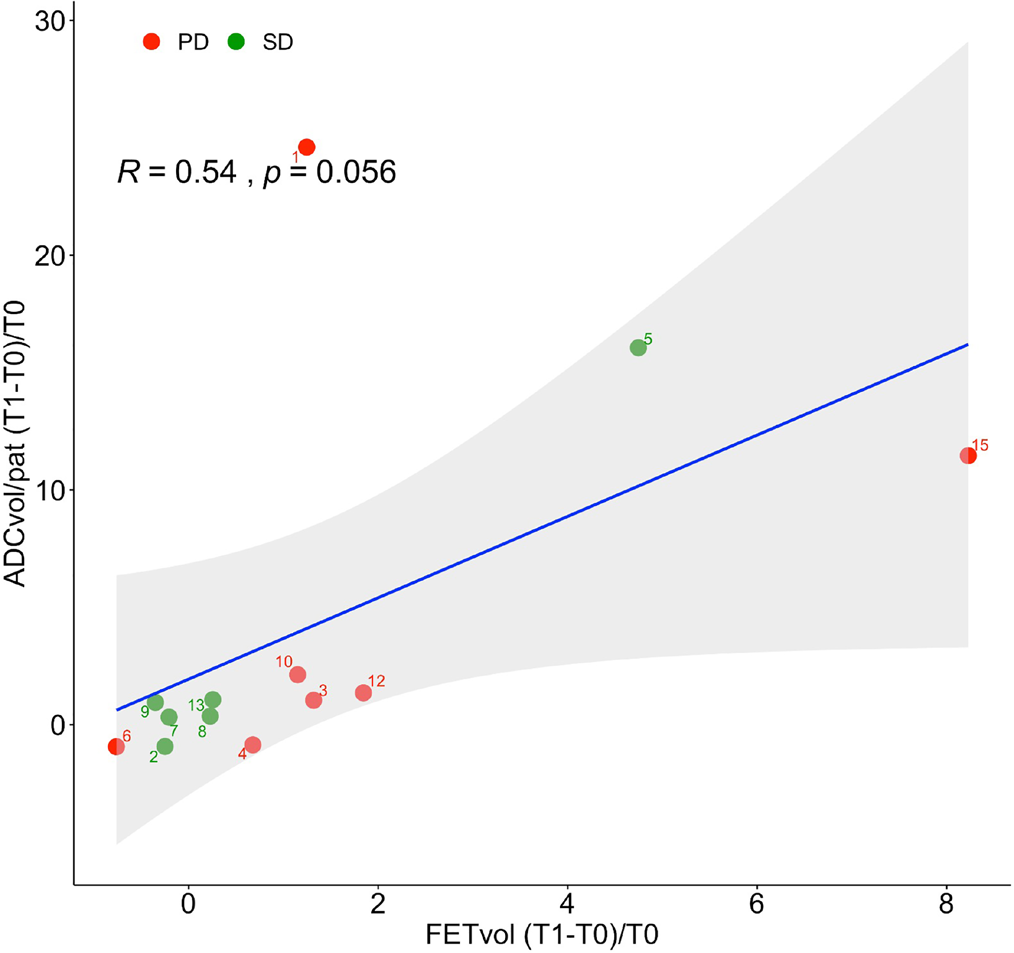
Scatter plots of the variation in pathological ADC volume (ADC_vol/pat_) *vs* the corresponding pathological FET volume (FET_vol/pat_) after two cycles of treatment with regorafenib. Red and green dots represent the patients presenting with PD and SD after regorafenib, respectively. The regression line is shown in blue, and the gray area represents the 95% confidence interval. ADC, apparent diffusion coefficient; SD, Stable Disease; PD, Progressive Disease; T0, First [^18^F]FET PET/MR; T1, Second [^18^F]FET PET/MR.

**Figure 4.**
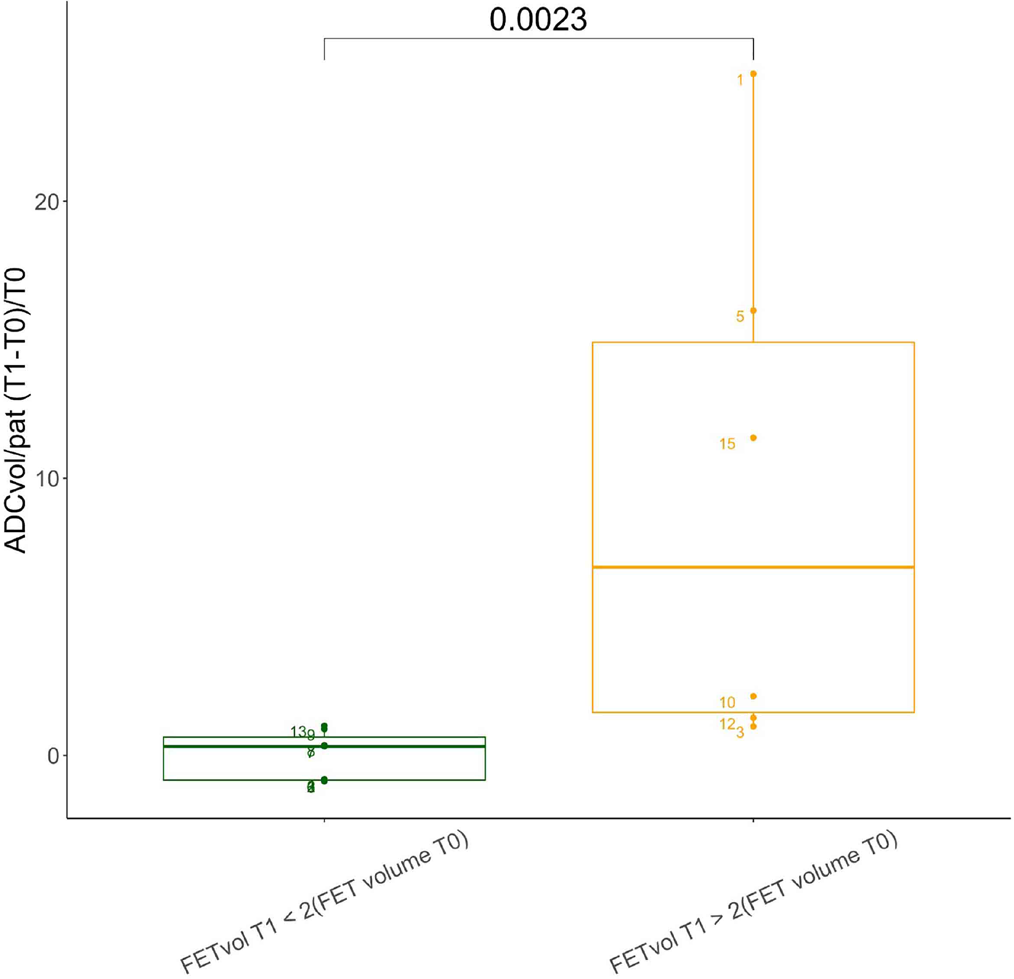
Box plots of the variation in the pathological ADC volume (ADC_vol/pat_) after two cycles of regorafenib. Study patients were subdivided into two groups: one including patients with at least a duplication in FET_vol/pat_ after treatment with regorafenib (in yellow), the other including subjects with a less than twofold increase in FET_vol/pat_ (in green). ADC, apparent diffusion coefficient; T0, First [^18^F]FET PET/MR; T1, Second [^18^F]FET PET/MR

After two cycles of regorafenib, 7/16 (44%) patients were observed to have SD, and the remaining 9/16 (56%) to have PD according to the RANO criteria (Table 2). The values of the [^18^F]FET PET/MR-derived parameters before and after treatment with regorafenib (FET_vol/pat_, TBR_mean_, TBR_max_,^23^ ADC_vol/pat_, and mean ADC_vol/pat_) are listed in Table 2 and summarized in Table 3. Their absolute and percentage variations after treatment are presented in Table 4. Although the absolute and percentage increases in FET_vol/pat_ were on average higher in PD than SD patients (21,605 mm^3^ and 168% *vs* −1160 mm^3^ and 70%), the differences between the two groups were not statistically significant (*p* = 0.17). Similarly, the average absolute and percentage increases in ADC pathological volume were also higher in PD than SD subjects (501 mm^3^ and 554% *vs* 33 mm^3^ and 297%), and also failed to reach statistical significance (*p* = 0.53). The percentage variations in mean ADC, FET_vol/pat_, TBR_max_ and TBR_mean_ did not differ significantly between SD and PD patients (Tables 3 and 4, Figure 2). When the percentage difference in FET pathological volumes was plotted against the corresponding percentage difference in ADC pathological volumes, a linear regression model revealed a correlation between the two variables (*R* = 0.54) (Figure 3). We found no evident correlation between the percentage variation in mean ADC values and the corresponding percentage variation in FET_vol/pat_ (*R* = 0.04). Patients with at least a twofold increase in FET pathological volume (Figure 4) after regorafenib showed a significantly higher increase in ADC pathological volume than the remaining subjects (*p* = 0.0023). In 2/9 subjects classified as progressive (according to RANO) after two cycles of regorafenib, the FET pathological volume decreased by 76 and 31%, respectively. Consistent with this, a decrease in the ADC pathological volume was observed in the former (−93%), while no residual pathological ADC areas could be detected in the latter. In contrast, in 3/7 patients classified as stable (according to RANO) after treatment, an increase in FET pathological volume (5895 mm^3^, 2057 mm^3^, and 1009 mm^3^, respectively) was observed; in the same patients, the ADC pathological volume increased at similar rates (273 mm^3^, 88 mm^3^, and 425 mm^3^).

**Table 2.** [^18^F ]FET PET/MR parameters at baseline and after two cycles of regorafenib. FET_vol/pat_ and ADC_vol/pat_ are the pathological segmented FET and ADC volumes, expressed in mm^3^; TBR_mean_ (mean Tumor-to-Background Ratio) and TBR_max_ (maximum Tumor-to-Background Ratio) were calculated as the mean and maximum uptakes, respectively, of the FET-positive area normalized for the mean background uptake

Baseline [18F]FET PET/MR						Post-Regorafenib [18F]FET PET/MR					
PT	FETvol/pat	TBRmean	TBRmax	ADCvol/pat	mean ADCvol/pat	FETvol/pat	TBRmean	TBRmax	ADCvol/pat	mean ADCvol/pat	RANO
**1**	4617	1.97	3.12	15	586.3	1,0370	1.99	3.28	384	604.7	PD
**2**	1,7322	2.07	3.50	913	563.0	1,3009	2.18	4.14	69	579.0	SD
**3**	3699	1.69	2.18	627	666.7	8578	1.78	2.55	1280	689.7	PD
**4**	9785	1.88	3.09	1252	645.5	1,6413	2.07	3.43	178	733.9	PD
**5**	1242	1.83	2.33	17	668.1	7137	2.11	3.67	290	687.1	SD
**6**	1,0710	1.79	3.42	631	657.9	2548	1.69	2.11	41	637.8	PD
**7**	1873	1.79	2.35	253	667.7	1486	1.82	2.39	335	646.3	SD
**8**	9052	1.79	2.47	240	383.2	1,1109	1.79	2.77	328	566.1	SD
**9**	6173	1.99	3.50	184	626.1	4007	1.87	2.77	359	558.4	SD
**10**	3941	1.87	3.25	266	606.7	8475	1.94	3.20	833	644.3	PD
**11**	2,2115	1.98	3.44	/	/	6,0399	2.19	3.40	2579	640.1	PD
**12**	3,9114	2.04	4.13	666	402.1	11,1268	2.14	4.37	1571	461.5	PD
**13**	3955	1.87	2.88	398	677.3	4964	1.89	2.78	823	675.4	SD
**14**	5,7593	2.20	4.29	/	/	3,9486	2.00	3.11	/	/	PD
**15**	1,0754	1.94	3.64	234	606.2	9,9238	2.35	4.64	2916	643.5	PD
**16**	2,4646	2.12	3.97	/	/	1,4428	1.98	3.17	717	586.4	SD

ADC, apparent diffusion coefficient; PD, Progressive Disease; SD, Stable Disease.

In two of the study subjects at baseline and in one post-regorafenib no pixels remained after ADC normalization. The RANO criteria were used to sort the patients into response categories.

**Table 3.** Means ± standard deviations and ranges of the [^18^F ]FET PET/MR-derived parameters at baseline and after 2 cycles of regorafenib

Parameter	Baseline [18F]FET PET/MR		Post-regorafenib [^18^F]FET PET/MR	
**FET_vol/pat_**	14161 ± 15387 mm^3^	1242–57593 mm^3^	25807 ± 34548 mm^3^	1486–111268 mm^3^
**TBR_max_**	3.22 ± 0.65	2.18–4.29	3.24 ± 0.70	2.11–4.64
**TBR_mean_**	1.93 ± 0.14	1.69–2.2	1.99 ± 0.18	1.69–2.35
**ADC_vol/pat_**	438 ± 363 mm^3^	15–1252 mm^3^	847 ± 887 mm^3^	41–2916 mm^3^
**Mean ADC _vol/pat_**	597 ± 97 * 10^−6^ mm^2^/s	383–677 * 10^−6^ mm^2^/s	624 ± 67 * 10^−6^ mm^2^/s	462–734 * 10^−6^ mm^2^/s

ADC, apparent diffusion coefficient; FET, O-(2-^18^F-fluoroethyl)-L-tyrosine; TBR, Tumor-to-Background Ratio.

FET_vol/pat_ and ADC_vol/pat_ are the pathological segmented FET and ADC volumes, expressed in mm^3^; TBR_mean_ (mean Tumor-to-Background Ratio) and TBR_max_ (maximum Tumor-to-Background Ratio) were calculated as the mean and maximum uptakes, respectively, of the FET-positive area normalized for the mean background uptake; mean ADC_vol/pat_ is the mean ADC value in the ADC_vol/pat_ volume.

**Table 4.** Variations in the [^18^F ]FET PET/MR-derived parameters in the subjects grouped according to RANO response category

Parameter	SD		PD	
	Mean ± standard deviation	Range	Mean ± standard deviation	Range
**ΔFET_pat/vol_**	−1160 ± 5138 mm^3^	−10218–5895 mm^3^	21605 ± 36769 mm^3^	−18107–88484 mm^3^
**ΔFET_pat/vol_ (%**)	70±199%	−41–474 %	168±261%	−76–823 %
**ΔTBR_max_**	0.1 ± 0.76	−0.8–1.34	−0.05 ± 0.74	−1.31–1
**ΔTBR_max_ (%**)	5 ± 29	−21–58	0 ± 21	−38–27 %
**ΔTBR_mean_**	0.03 ± 0.14	−0.14–0.28	0.09 ± 0.18	−0.2–0.41
**ΔTBR_mean_ (%**)	1±8%	−6–15 %	5±9%	−9–21 %
**ΔADC_pat/vol_**	33 ± 448 mm^3^	−844–425 mm^3^	501 ± 1200 mm^3^	−1074–2682 mm^3^
**ΔADC_pat/vol_ (%**)	297±645%	−92–1606 %	554±940%	−94–2460 %
**ΔMean ADC_pat/vol_**	21 ± 85 * 10^−6^ mm^2^/s	−67–182 * 10^−6^ mm^2^/s	35 ± 34 * 10^−6^ mm^2^/s	−20–88 * 10^−6^ mm^2^/s
**ΔMean ADC_pat/vol_ %**	6±21%	−11–48 %	6±6%	−3–15 %

ADC, *apparent diffusion coefficient*; FET, *O-(2-18F-fluoroethyl)-L-tyrosine*; PD, Progressive Disease; SD, Stable Disease;TBR, *Tumor-to-Background Ratio*.

FET_vol/pat_ and ADC_vol/pat_ are the pathological segmented FET and ADC volumes; TBR_mean_ is the mean Tumor-to-Background Ratio and TBR_max_ is the maximum Tumor-to-Background Ratio; mean ADC_vol/pat_ is the mean ADC value in the ADC_vol/pat_ volume. RANO criteria were used to sort the patients into response categories.

The Kaplan–Meier analysis showed that the percentage variations of FET_PAT/VOL,_ ADC_PAT/VOL_ and RANO criteria were able to predict overall survival (*p* = 0.02, *p* = 0.024 and *p* = 0.04 respectively). TBR Max and TBR mean were not able to accurately predict overall survival.

## Discussion

One of the main finding to emerge from the present study was the correlation between the percentage changes in the pathological FET and ADC volumes in recurrent GBM patients treated with regorafenib at their first disease relapse. To our knowledge, this is the first work assessing the variation in the ADC signal in the FET-positive volume in patients undergoing [^18^F]FET PET/MR both at baseline and soon after beginning this new beneficial second-line therapy. The value of DWI-derived parameters in treatment monitoring of GBM patients has already been extensively investigated,^32–35^ and many authors have suggested that the DWI methodology could play an important role in guiding response assessment, particularly when conventional contrast-enhanced and *T*_2_ weighted/FLAIR sequences are less reliable. Although DWI sequences are routinely acquired as part of the standard MR protocol for brain tumor imaging, the most recent recommendations^36^ only describe how diffusion-weighted images should be acquired and provide no guidance for clinically interpreting and quantifying the extent of the tumor for the purpose of response evaluation. Two major issues consequently arise, the first regarding the strategy to identify the region on the DWI-ADC images to be analyzed, the second regarding the threshold for pathological ADC values. In most of the published studies, the tumor volume was outlined and the VOI constructed on contrast-enhanced *T*_1_ weighted images, which were subsequently transferred to the corresponding DWI-ADC images. Buemi et al,^37^ *e.g.* manually drew the VOIs encompassing the areas of tumor-related contrast enhancement, and *T*_2_ weighted/FLAIR abnormalities were mapped onto the corresponding ADC images, thus deriving the CE-ADC and T2/FLAIR-ADC volumes, respectively. Histogram analysis and curve fitting using a two-mixture normal distribution model were carried out to calculate the mean ADC of the lowest ADC values in these areas (CE-ADC-L and T2/FLAIR-ADC-L).^37^ Interestingly, only the mean ADC in CE-ADC-L turned out to be significantly predictive of progression-free survival and overall survival in GBM patients treated with bevacizumab and fotemustine. The predictive value of the low-ADC areas is confirmed by other published papers.^38,39^ Zeiner et al^40^ calculated the ADC-ratio by measuring the minimum ADC values in the tumor and normalizing them by the ADC values of the contralateral, normal appearing brain tissue. In our study, instead, a standard VOI contralateral to the lesion was used to determine the appropriate threshold for the selection of pathological ADC values. This approach allowed for a more direct identification of the low ADC values in the defined VOIs, avoiding the need for complex mathematical models. However, the post-regorafenib variation in the mean ADC thus calculated did not significantly correlate with the corresponding change in FET-positive volume, nor with the RANO response categories. A possible explanation for this discrepancy may lie in the different methodological approaches used here and in the previously published studies.^12,37–48^ This highlights the importance of future efforts towards standardizing the analysis of ADC maps before considering the inclusion of this methodology in the response assessment criteria.

It is important to note that the changes in neither the pathological FET volume nor the pathological ADC volume were significantly different in the stable and progressive patients as assessed by RANO criteria. RANO criteria are based mostly on changes in T2/FLAIR and contrast-enhanced areas, which are known to be affected by edema, inflammation, gliosis, and disruption of the blood–brain barrier. DWI, instead, is sensitive to microscopic water motion, resulting in relatively restricted diffusion in areas of tightly packed tumor cells. However, diffusion may be altered by causes other than increased cellularity in neoplastic tissue, and the diagnostic performance of this methodology is influenced by the choice of the appropriate DWI parameter to analyze.^16,49,50^ Moreover, the heterogeneity of the ADC signal may have translated into the wide variability we observed in the changes in the pathological ADC volume after regorafenib. This, in turn, may explain why a relatively high threshold of increase in FET pathological volume was needed to subdivide our population into groups with significantly different pathological ADC volumes.

**Figure 5.**
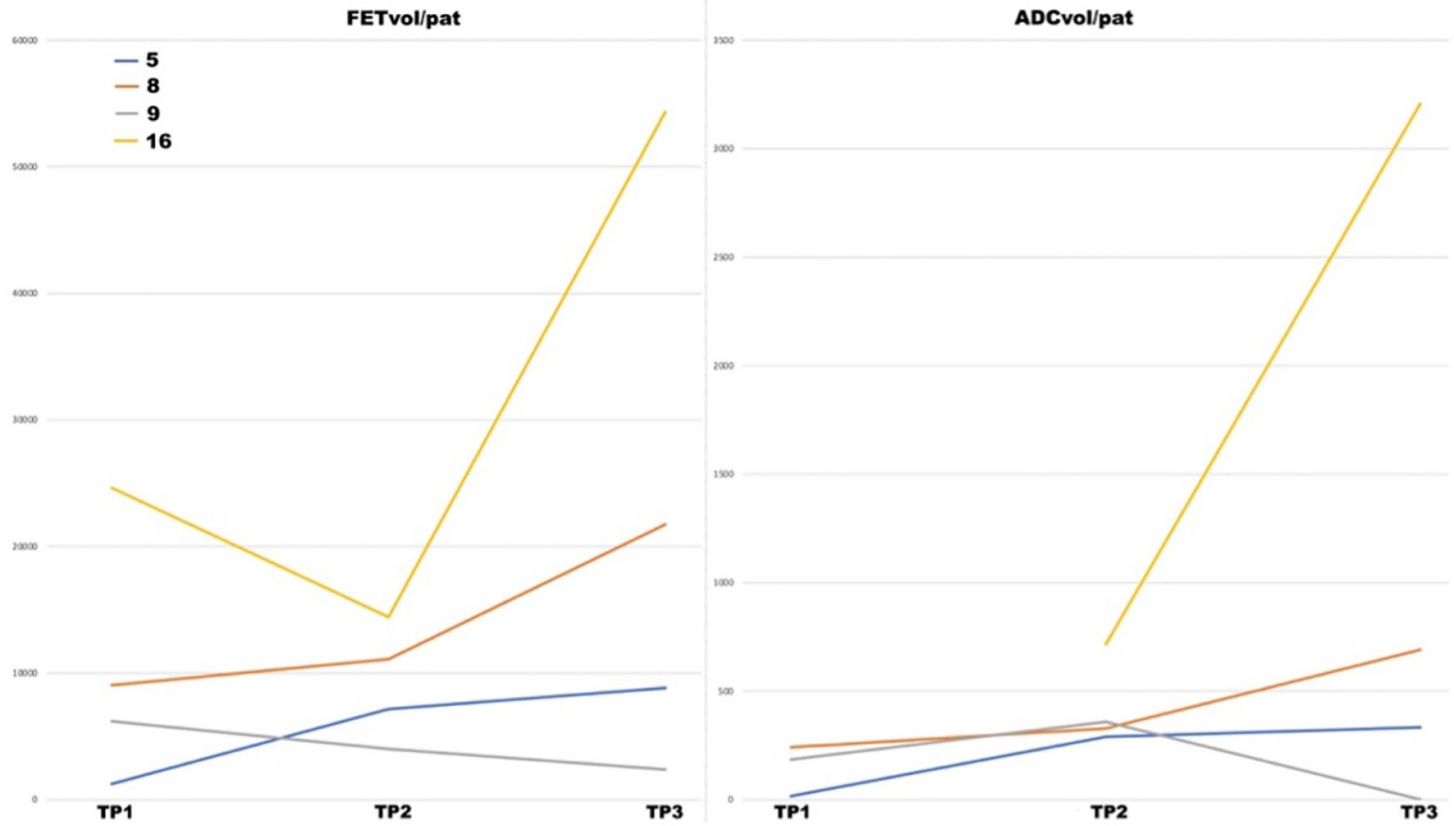
Variations in the pathological FET volume (FET_vol/pat_) and pathological ADC volume (ADC_vol/pat_) between timepoints (TP1, TP2 and TP3 representing, respectively, the first baseline PET/MR before regorafenib, the second PET/MR after two cycles of regorafenib, and the third PET/MR after six cycles of regorafenib). ADC, apparent diffusion coefficient; FET, *O-*(2-18F-fluoroethyl)-L-tyrosine; PET, positron emission tomography; TP1, Timepoint 1; TP2, Timepoint 2; TP3 = Timepoint 3.

**Figure 6.**
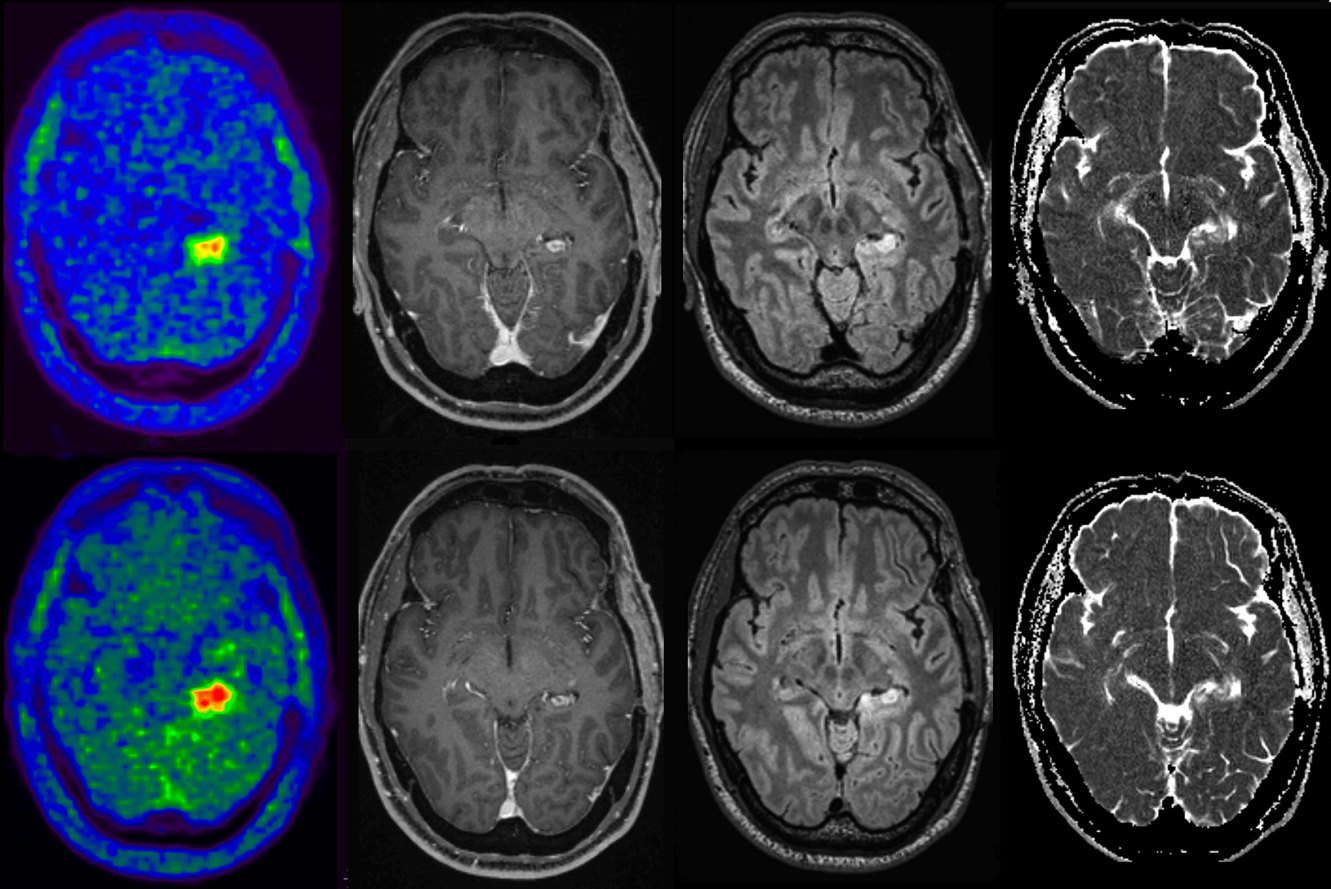
Axial [^18^F]FET PET/MR images before (upper row) and after (lower row) treatment with regorafenib in one of the study patients (#5). first column: [^18^F]FET PET; second column: post-contrast *T*_1_ weighted; third column: FLAIR; fourth Column: ADC maps. The increase in [^18^F]FET uptake after treatment with regorafenib is evident in lower row first column compared with upper row even though no significant increases in the contrast-enhanced and FLAIR altered areas are visible. The analysis revealed an increase in the pathological ADC-volume after regorafenib, even though it was barely detectable qualitatively on the native images. ADC, apparent diffusion coefficient; FET, *O-*(2-18F-fluoroethyl)-L-tyrosine; FLAIR, fluid attenuated inversion recovery; PET, positron emission tomography.

In three cases which were classified, according to the RANO criteria, as stable (SD) after treatment with regorafenib, both the ADC and FET pathological volumes increased compared with the baseline examinations (patients #5, #8 in Figure 5, and #13). Information from subsequent follow-ups was available for two of these patients: a) patient #5 (interestingly, classified as SD by the RANO criteria) showed a slight increase in FET_vol/pat_ (and to a lesser extent also in ADC_vol/pat_) at a PET/MR examination (Figure 6) performed 2 months later (TP3 in Figure 5), and presented disease progression at an MR scan performed 4 months later; b) patient #8 showed a significant increase in FET_vol/pat_ (and to a lesser extent also in ADC_vol/pat_) at a subsequent PET/MR examination (TP3 in Figure 5), and was consistently considered progressive according to the RANO criteria. We were able to carry out a follow-up PET/MR in another two cases (patients #9 and #16, both SD at the PET/MR examination after two cycles of regorafenib): a) patient # presented minimal variations in FET_vol/pat_ and ADC_vol/pat_ after two cycles of regorafenib, and remained stable (presenting a decrease in FET_vol/pat_ and ADC_vol/pat_) at the follow-up PET/MR (TP3 in Figure 5); b) patient #16 showed a significant increase in FET_vol/pat_ and ADC_vol/pat_ between the PET/MR performed after two cycles of regorafenib (Figure 5) and the follow-up PET/MR, and was then considered progressive according to the RANO criteria.

These four cases, although insufficient to draw definitive conclusions, seem to show consistent variations in FET and ADC pathological volumes in follow-up examinations performed after six cycles of regorafenib, and seems to confirm the greater predictive value of these parameters compared with the standard RANO criteria. In fact, the variations in FET and ADC (in the PET/MR after two cycles of regorafenib) predicted the follow-up in two out of four cases wrongly classified by RANO (subjects #5 and #8 who were categorized as stable according to the RANO criteria).

**Figure 7.**
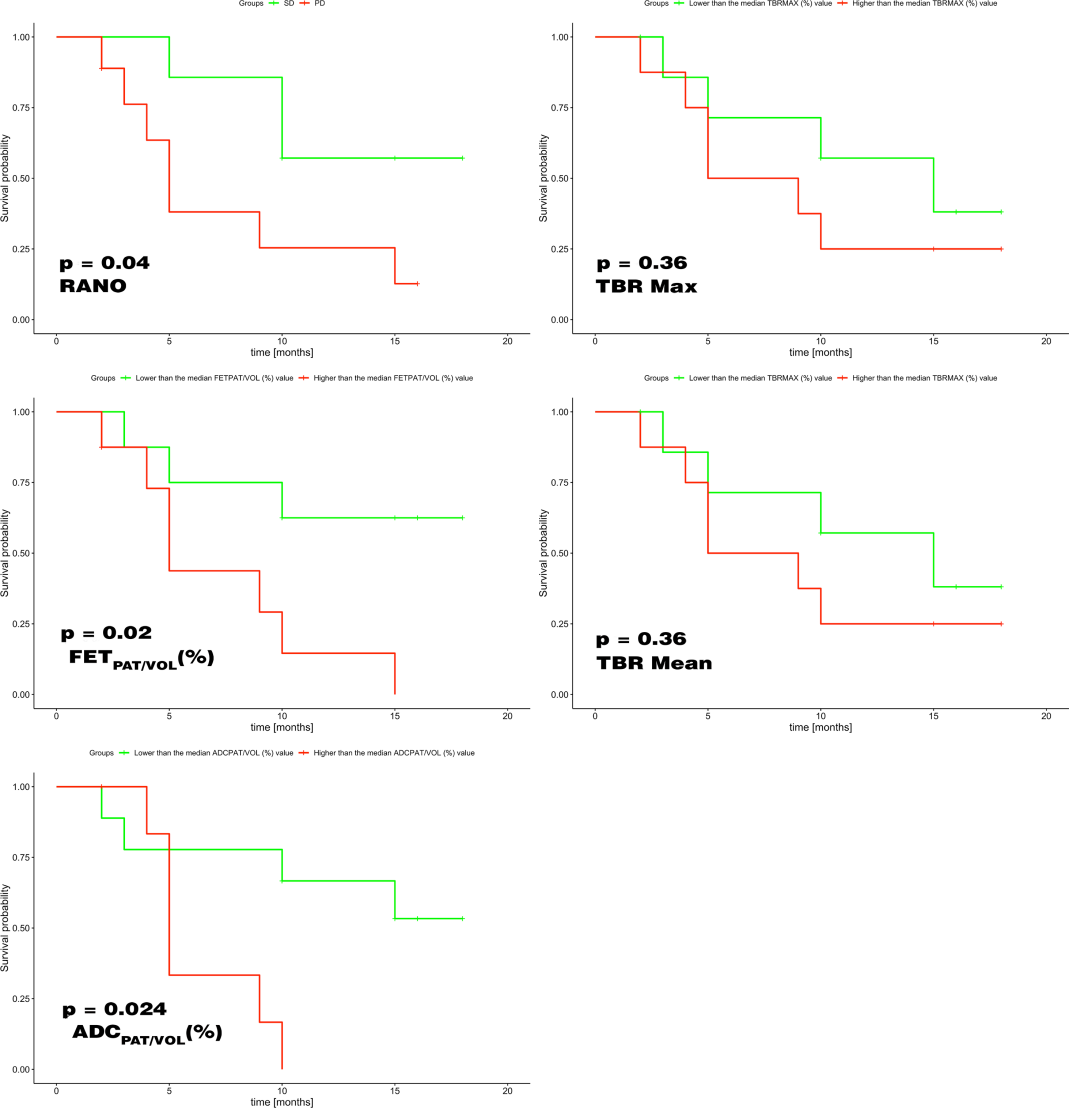
Kaplan–Meier analysis shows that the percentage variations of FET_PAT/VOL_ and ADC_PAT/VOL_ performed at least as well as RANO criteria or even better in terms of overall survival prediction (left column). TBR Max and TBR mean (right column) on the other hand are not able to accurately predict overall survival. ADC, apparent diffusion coefficient; FET, *O-*(2-18F-fluoroethyl)-L-tyrosine; TBR, Tumor-to-Background Ratio.

Kaplan–Meier analysis (Figure 7), performed to compare the performance in overall survival prediction, revealed that the percentage variations of FET_PAT/VOL_ and ADC_PAT/VOL_ performed at least as well as RANO criteria (*p* = 0.02, *p* = 0.024 and *p* = 0.04 respectively) or even better. TBR Max and TBR mean on the other hand, frequently used at the first tumor occurrence, are not able to accurately predict overall survival.

Therefore, the so identified [^18^F]FET and ADC areas and values, which are correlated but were obtained from completely different measures, could serve as independent biomarkers of treatment response and could, at least, complement the RANO criteria especially in doubtful cases.

Our study has some limitations, including that regorafenib was introduced only recently:

1. It was retrospective in nature and included only a relatively (considering the actual infrequent use of the treatment) small number of patients.
2. The current RANO criteria were assumed as gold-standard.

Despite these limitations, we focused on a highly homogeneous patient population comprising GBM subjects at their first disease relapse, and all patients were treated with a recently approved chemotherapeutic agent (regorafenib). Moreover, all imaging studies were acquired at the same institution with an integrated PET/MR system and a standardized protocol.

## Conclusions

In the present study, we have proposed a method to identify the pathological ADC volume based on the corresponding [^18^F]FET positive region in intrinsically co-registered [^18^F]FET PET/MR images. We found a correlation between the percentage changes in pathological FET and DWI-ADC volumes in glioblastoma patients treated with regorafenib at their first disease relapse. In 4/16 cases followed up with a third PET/MR, the results seemed encouraging compared to the RANO criteria.

Kaplan analysis showed that FET_PAT/VOL_ and ADC_PAT/VOL_ performed at least as well as RANO criteria in terms of overall survival prediction.

The [^18^F]FET and ADC metrics identified could, given they were correlated but obtained from completely different measures, serve as semi-quantitative independent biomarkers of response to regorafenib treatment.
